# Global sequence variation in the histidine-rich proteins 2 and 3 of *Plasmodium falciparum: *implications for the performance of malaria rapid diagnostic tests

**DOI:** 10.1186/1475-2875-9-129

**Published:** 2010-05-17

**Authors:** Joanne Baker, Mei-Fong Ho, Anita Pelecanos, Michelle Gatton, Nanhua Chen, Salim Abdullah, Audrey Albertini, Frederic Ariey, John Barnwell, David Bell, Jane Cunningham, Djibrine Djalle, Diego F Echeverry, Dionicia Gamboa, Jeffery Hii, Myat Phone Kyaw, Jennifer Luchavez, Christopher Membi, Didier Menard, Claribel Murillo, Sina Nhem, Bernhards Ogutu, Pamela Onyor, Wellington Oyibo, Shan Qing Wang, James McCarthy, Qin Cheng

**Affiliations:** 1 Department of Drug Resistance and Diagnostics, Australian Army Malaria Institute, Brisbane, Australia; 2 Clinical Tropical Medicine Laboratory, Queensland Institute of Medical Research, University of Queensland, Herston, Australia; 3 Malaria Drug Resistance and Chemotherapy Laboratory, Queensland Institute of Medical Research, Herston, Australia; 4 Bagamoyo/Ifakara Health Research and Development Centre, Ifakara, United Republic of Tanzania; 5 Foundation for Innovative and New Diagnostics, Geneva, Switzerland; 6 Pasteur Institute of Cambodia, Phnom Penh, Cambodia; 7 Centre for Disease Control and Prevention, Atlanta, USA; 8 Global Malaria Programme, World Health Organization, Geneva, Switzerland; 9 UNICEF/UNDP/World Bank/WHO Special Programme for Research and Training in Tropical Diseases (TDR), Geneva, Switzerland; 10 Institut Pasteur de Bangui, Bangui, Central African Republic; 11 Centro Internacional de Entrenamiento e Investigaciones Medicas (CIDEIM), Cali, Colombia; 12 Instituto de Medicina Tropical Alexander Von Humboldt, Universidad Peruana Cayetano Heredia, Peru; 13 Departamento de Bioquimica, Biologia Molecular y Farmacologia, Facultad de Ciencias y Filosofia, Universidad Peruana Cayetano Heredia, Peru; 14 Western Pacific Regional Office of the World Health Organization, Solomon Islands; 15 Department of Medical Research (Lower Myanmar), Yangon, Myanmar; 16 Research Institute for Tropical Medicine, Alabang, The Philippines; 17 Institut Pasteur de Madagascar, Madagascar; 18 Centre for Clinical Research, Kenya Medical Research Institute, Kisumu, Kenya; 19 College of Medicine, University of Lagos, Odoaraba, Lagos, Nigeria; 20 Hainan Provincial Centre for Disease Control and Prevention, Haikou, Hainan, China; 21 School of Population Health, University of Queensland, Herston, Queensland, Australia

## Abstract

**Background:**

Accurate diagnosis is essential for prompt and appropriate treatment of malaria. While rapid diagnostic tests (RDTs) offer great potential to improve malaria diagnosis, the sensitivity of RDTs has been reported to be highly variable. One possible factor contributing to variable test performance is the diversity of parasite antigens. This is of particular concern for *Plasmodium falciparum *histidine-rich protein 2 (PfHRP2)-detecting RDTs since PfHRP2 has been reported to be highly variable in isolates of the Asia-Pacific region.

**Methods:**

The *pfhrp2 *exon 2 fragment from 458 isolates of *P. falciparum *collected from 38 countries was amplified and sequenced. For a subset of 80 isolates, the exon 2 fragment of histidine-rich protein 3 (*pfhrp3*) was also amplified and sequenced. DNA sequence and statistical analysis of the variation observed in these genes was conducted. The potential impact of the *pfhrp2 *variation on RDT detection rates was examined by analysing the relationship between sequence characteristics of this gene and the results of the WHO product testing of malaria RDTs: Round 1 (2008), for 34 PfHRP2-detecting RDTs.

**Results:**

Sequence analysis revealed extensive variations in the number and arrangement of various repeats encoded by the genes in parasite populations world-wide. However, no statistically robust correlation between gene structure and RDT detection rate for *P. falciparum *parasites at 200 parasites per microlitre was identified.

**Conclusions:**

The results suggest that despite extreme sequence variation, diversity of PfHRP2 does not appear to be a major cause of RDT sensitivity variation.

## Background

Malaria is one of the most important infectious diseases of humanity, and continues to cause significant mortality and morbidity worldwide. Early diagnosis is important for case management and treatment of the disease, and in guiding treatment for non-malaria fevers. Symptom-based clinical diagnosis is inaccurate, and contributes to poor management of febrile illness, over-treatment of malaria, and may promote drug resistance to current anti-malarials [1].

Rapid diagnostic tests (RDTs) for malaria have the potential to improve case management and thereby reduce morbidity and mortality, especially in remote areas, facilitating the timely delivery of appropriate treatment. Indeed, many RDTs today can achieve excellent sensitivity and specificity for *Plasmodium falciparum *at a parasitaemia greater than 500 parasites per microlitre (parasites/μL) [2]. At lower parasitaemia, however, variability in sensitivity is more common [3-7].

Today, over 150 malaria RDTs are commercially available, with most using a *P. falciparum *detecting component targeting *P. falciparum *histidine-rich protein 2 (PfHRP2). The gene encoding the protein, *pfhrp2*, is a single copy subtelomeric gene located on chromosome 7 encoding an amino acid sequence containing 34% histidine, 37% alanine and 10% aspartic acid [8-11]. PfHRP2 is characterized by multiple contiguous repeats of the sequences AHH and AHHAAD [8,9]. PfHRP2 is a 60-105 kD water-soluble protein specific to *P. falciparum*, synthesized and present throughout the asexual life cycle, identified as a surface-exposed protein in infected erythrocytes [8-10,12-18]. The protein is also found circulating in the peripheral blood of infected individuals [19]. These features make PfHRP2 a good target for diagnosis of *P. falciparum *infection.

*Pfhrp3 *encodes *P. falciparum *histidine-rich protein 3 (PfHRP3), also known as the small histidine-rich protein (SHARP), located near one end of chromosome 13 [17,20]. *Pfhrp3 *shares many structural similarities with *pfhrp2*. Both genes have an interrupted structure and contain a signal peptide sequence in exon 1 followed by an intron. The intron is followed by the main coding region, exon 2. Exon 2 in both *pfhrp2 *and *pfhrp3 *encodes histidine-rich amino acid repeats beginning 75-90 nucleotides downstream from its start [18]. Although the histidine composition of PfHRP3 is slightly less than that of PfHRP2 (28% compared to 34%), both genes share many histidine and alanine rich repeats [10]. It has been suggested that, due to their similarity, both genes are related, derived from an ancestral duplication and interchromosomal divergence from a common ancestral gene, and may complement each other in function [8,10,17,18]. Antibodies against PfHRP2 cross-react with PfHRP3 [8,18]. Thus, PfHRP3 also contributes to the detection of *P. falciparum *infections in PfHRP2-detecting malaria RDTs.

As part of the World Health Organization (WHO) and Foundation for Innovative and New Diagnostics (FIND) Malaria RDT Quality Assurance Programme, the levels of diversity for antigens targeted by malaria RDTs have been systematically investigated. While parasite aldolase and pLDH appear to be highly conserved [21-23], *pfhrp2 *was found to be highly variable. In the preliminary analysis of 74 isolates from mostly Southwest Pacific and Asian countries, a significant sequence variation in *pfhrp2 *and *pfhrp3 *was observed in isolates within the same country and between different countries [24]. This raised a serious concern that the sequence variation could result in significant variation in the presence and frequency of epitopes recognized by monoclonal antibodies (MABs) and hence impact on the RDT detection sensitivities for different parasites. This concern was strengthened by a regression analysis based on 16 cultured parasite lines tested where the number of type 2 (AHHAHHAAD) and type 7 (AHHAAD) repeats in PfHRP2 were identified to be a contributing factor to the variable sensitivity reported at low level parasitaemia (below 250 parasites/μl) [24,25]. While this established extensive variation and a preliminary link between sequence diversity and RDT sensitivity within this sample set, it did not comprehensively cover diversity from all areas of global malaria transmission or testing sensitivity on a large set of samples with a wide range of RDT products.

The aims of the current study were to extend the diversity investigation to include isolates from African and South American countries, and to better understand the implication of global diversity in *pfhrp2 *and *pfhrp3 *on the performance of PfHRP2- detecting RDTs. In this paper, the global diversity of *pfhrp2 *and *pfhrp3 *was examined and the distribution of variants mapped. Furthermore, the results of the recently completed WHO product testing of malaria RDTs: Round 1 (2008) [26] were used to examine the effect of PfHRP2 sequence structure on RDT sensitivity.

## Methods

### Parasite lines and isolates

Field isolates of *P. falciparum *were obtained from patients and preserved on filter paper, and laboratory lines were cultured at the Australian Army Malaria Institute (AMI). Patient blood sample collection was coordinated by WHO, TDR and FIND and conducted by investigators and institutions within the WHO-FIND Malaria RDT Quality Assurance Programme. Protocols were approved by each collection country's Ethics Review Board and the WHO Research Ethics Review Committee. Fingerprick blood samples were obtained from consenting individuals, and three drops of patient blood were collected onto Whatman Filter paper (Grade 1, 9.0 cm, Whatman International Ltd, Maidstone, England) and air-dried for storage. All filter papers were sent to AMI for processing which was approved by the Australian Defence Human Research Ethics Committee (ADHREC 377/05). Confirmation of parasitaemia was obtained by microscopy for each patient and was also conducted for cultured lines. The 458 samples used to examine *pfhrp2 *(including 74 reported in [24]) and their country of origins are detailed in Table 1. The subset of 80 samples that was used to examine *pfhrp3 *is also listed in Table 1.

**Table 1:** Country Origins (ISO code) and number *Plasmodium falciparum *isolates sequenced for *prfhp2 *and *pfhrp3 *and the number of different *pfhrp2 *sequence types.

Region/Country	*pfhrp2*		*pfhrp3*		Region/Country	*pfhrp2*		*pfhrp3*	
			
	*n*	No. types	*n*	No. types		*N*	No. types	*n*	No. types
AFRICA					CENTRAL & SOUTH AMERICA				
Benin (BJ)	1	1			Brazil (BR)	9	6	2	1
Burkina Faso (BF)	1	1			Colombia (CO)	12	8	1	1
Cameroon (CM)	2	2			Ecuador (EC)	1	1		
Central African Republic (CF)	13	13	1	1	Haiti (HT)	10	6	4	4
Gambia (GM)	1	1	1	1	Honduras (HN)	3	2		
Ghana (GH)	6	6			Peru (PE)	18	4	6	2
Guinea (GN)	1	1			Santa Lucia (LC)	1	1		
Kenya (KE)	30	30	6	6	Suriname (SR)	3	1	2	1
Liberia (LR)	3	3			*Sub total*	*57*		*15*	
Madagascar (MG)	17	17			ASIA				
Malawi (MW)	2	2			Cambodia (KH)	32	24	1	1
Nigeria (NG)	80	74	16	11	China (CN)	10	7	4	3
Niger (NE)	1	1			Indonesia (ID)	5	4		
Sierra Leone (SL)	2	2	1	1	Malaysia (MY)	2	2	1	1
Sudan (SD)	3	3			Myanmar (MM)	5	5		
Tanzania (TZ)	39	34	2	2	Philippines (PH)	45	27	7	4
Uganda (UG)	2	2			Thailand (TH)	7	6	4	4
Zambia (ZM)	2	2			Vietnam (VN)	5	4		
*Sub total*	*206*		*27*		*Sub total*	*111*		*17*	
SOUTHWEST PACIFIC									
Papua New Guinea (PG)	17	12	7	6					
Solomon Is. (SB)	35	17	13	9					
East Timor (TP)	24	12							
Vanuatu (VU)	8	4	1	0					
*Sub total*	*84*		*21*						
**Global total**	458		80						

### DNA isolation, PCR amplification and sequencing of *pfhrp2 *and *pfhrp3*

Parasite DNA was extracted, from both patient blood preserved on filter paper and from packed red cells of cultured isolates, using the QIAgen QiaBlood kit (QIAGEN, Germany) following the manufacturer's instructions. Using the same primers and PCR conditions described previously [24], close to full length exon 2 of the *pfhrp2 *(+65bp to stop codon) and *pfhrp3 *(+56bp to 1bp before stop codon) genes were amplified and sequenced (ABI), and the nucleotide sequence translated to corresponding amino acids (aa).

### Translation of DNA sequences and analysis of repeats

The same bar code system as previously reported was used to ascertain the repeat types present in *pfhrp2 *and *pfhrp3 *[24]. Six additional codes were described, and were designated types 19-24. These newly identified repeats are detailed in Table 2. The sequences reported in this article have been deposited in the GenBank database (GenBank accession numbers for *pfhrp2 *FJ871160 to FJ871401; GenBank accession numbers for *pfhrp3 *GU194966 to GU195043). The sequences were aligned using Microsoft Excel software to examine similarity between isolates. Identical sequences were identified and assigned to the same grouping, while different sequences were listed separately.

**Table 2:** The presence (+) and absence (-) of amino acid repeats in PfHRP2 and PfHRP3.

Code	Repeat	PfHRP2	PfHRP3
1	AHHAHHVAD	+	+
2	AHHAHHAAD	+	+
3	AHHAHHAAY	+	-
4	AHH	+	+
5	AHHAHHASD	+	-
6	AHHATD	+	-
7	AHHAAD	+	+
8	AHHAAY	+	-
9	AAY	+	-
10	AHHAAAHHATD	+	-
11	AHN	+	-
12	AHHAAAHHEAATH	+	-
13	AHHASD	+	-
14	AHHAHHATD	+	-
15	AHHAHHAAN	-	+
16	AHHAAN	-	+
17	AHHDG	-	+
18	AHHDD	-	+
19	AHHAA	+	-
20	SHHDD	+	+
21	AHHAHHATY	+	-
22	AHHAHHAGD	+	-
23	ARHAAD	+	-
24	AHHTHHAAD	+	-

Repeat types not previously reported are underlined.

### Statistical analyses

Variability of repeat frequencies for *pfhrp2 *and *pfhrp3 *within and between isolates of different geographic origin was examined statistically, and observations made on differences in exon length, motif use, and frequency distribution. GraphPad PRISM^® ^(GraphPad Software, La Jolla, USA) and Statistical Package for the Social Sciences^® ^(SPSS Inc, Chicago, USA) were employed to analyse the sequence data for the following purposes:

1. Geographical differences in the sequence characteristics. Differences in the total number of amino acids and the number of repeats of each type, between countries (38 countries) and between regions (four different regions, grouped to Africa, Southwest Pacific, Asia and South America) were tested using the Kruskal-Wallis test. Tukey's test was used for post hoc multiple comparisons. To investigate whether a correlation existed between amino acid length of PfHRP2 and PfHRP3, we examined the relationship of total amino acid length for both genes.

Further analysis of *pfhrp2 *repeat types 2 and 7 and *pfhrp3 *repeat types 15, 16, 17 and 18, was conducted to look for individual country differences in the number of these repeats. The one sample t-test was used to determine whether there were significant differences between a given country's mean repeat number, compared to mean number for all countries combined.

2. Predictive value of sequences to RDT performance. The sequence characteristics for the 79 isolates used in the WHO Product Testing of Malaria RDTs (Round 1) were selected [26]. These geographically varying isolates had different *pfhrp2 *sequences and repeat structures, classified as type A (≥100), B (50-99), or C (< 50), according to the frequency of their type 2 × type 7 repeats [24]. Using only the results from the PfHRP2-detecting RDTs, the percent of the products testing positive to each parasite isolate at 200 parasites/μL was determined and linear regression used to investigate whether sequence structure and length influenced the detection rate.

## Results

### Variation in *pfhrp2*

The PCR product of *pfhrp2 *exon 2 varied markedly in size between different parasite isolates. The amplified exon 2 size within the entire set of 458 isolates/laboratory lines examined ranged from 561bp to 918bp, with an average of 783 bp. The size variation was largely attributed to variation in numbers of 27- and 18- bp repeats. Three hundred and eighteen different *pfhrp2 *sequences were identified consisting of combinations of 20 different amino acid repeats (Table 2). Two hundred and fifty nine isolates had a unique *pfhrp2 *sequence, while the remaining 59 sequences were seen in > 1 parasite isolate. Of these 59 sequences, 36 (61%) were shared by isolates from the same country, while the remaining 23 (39%) were common to isolates from different countries (Table 3), of which 10 (44%) were shared within the region and 13 (56%), between different regions.

**Table 3:** The country (by ISO code) distribution of shared *pfhrp2 *and *pfhrp3 *sequences.

*pfhrp2*					
**Seq. type**	**Shared in** **Country**	**Seq. type**	**Shared in** **Country**	**Seq. type**	**Shared in** **Country**

a	SB, PH	u	KE, TP, TH	ao	NG
b	ID, NG	v	CN	ap	CO, HT
c	PG, VU	w	SB	aq	CO, PE
d	UG, KH, MY, PH	x	BR, PE,	ar	GH, NG
e	SB, PH	y	HN, LC	as	TH
f	SB, PH	z	NG	at	CO, KH
g	PH	aa	LR, PG	au	NG
h	SD, SB	ab	BR	av	NG, TZ, PE, KH
i	SB	ac	PH	aw	KH
j	PH	ad	HT	ax	KH
k	PH	ae	PH	ay	KH, ID, MM
l	PH	af	TP	az	KH
m	VN	ag	BR	ba	CO
n	VU	ah	CN	bb	NG
o	PH	ai	CN	bc	PH
p	TP	aj	BR, SR	bd	NG, TZ
q	SB	ak	NG	be	PG, ID
r	TP	al	NG	bf	NG, SB
s	SB	am	PG, SB	bg	SB
t	GH, PH, TH	an	PE		

***pfhrp3***					

bh	PE	bn	CN, SR	bs	NG, SB
bi	KE, TH, CO, HT	bo	NG, CF, MY	bt	PG, SB, HT
bj	NG	bp	PG, SB	bu	PG, KH
bk	NG	bq	KE, PH	bv	NG, VU
bl	PH	br	PG, TH	bw	SB
bm	NG, CN, PE, BR				

**Figure 1:**
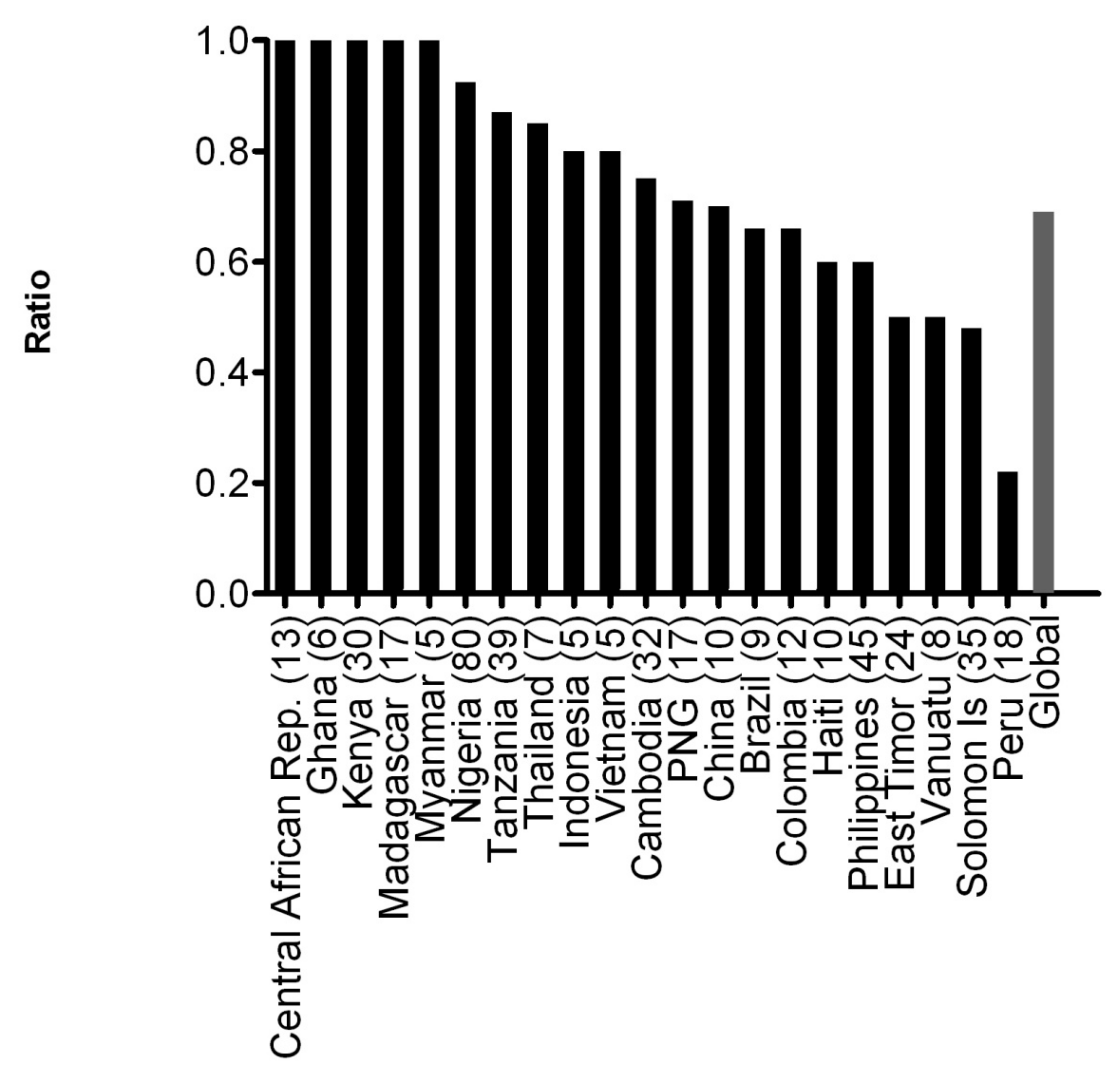
**Ratio of number of unique sequences to total sequences for *pfhrp2 *for countries with a minimum of 5 isolates**. Numbers in brackets indicate the number of samples

The number of different *pfhrp2 *sequence types observed in each country is listed in Table 1. To demonstrate the level of sequence diversity the ratio of the number of *pfhrp2 *sequence types to total number of sequences in a country was calculated for all countries. The overall ratio is 0.69 (318 unique sequence/458 isolates) with a unique *pfhrp2 *type observed in every 1.44 parasite isolates examined. The ratio varied between different countries. Figure 1 shows the range of ratios for countries with more than 5 *pfhrp2 *sequences analysed. Isolates from Peru (*n *= 18, ratio 0.22) showed the least *pfhrp2 *sequence variability among all countries sampled with the 18 isolates sharing only 4 different sequence types. The Solomon Islands and The Philippines showed medium range sequence diversity. Of the Solomon Island isolates (*n *= 35, ratio 0.48), 28 samples combined to give 10 different sequence types, often identical to sequences found in isolates from other countries. The remaining seven Solomon Island isolates each had a distinct unique sequence. The Philippines isolates (*n *= 45, ratio 0.6) showed slightly greater diversity, with the 32 sequences combining to give 14 unique sequences, and the remaining 13 isolates having unique sequences. The greatest variability in *pfhrp2 *sequence was found in the Central African Republic (*n *= 13), Ghana (*n *= 6), Kenya (*n *= 30), Madagascar (*n *= 17) and Myanmar (*n *= 5), where no sequence was identical to another either within the country (ratio = 1.0) or with any other country.

**Figure 2:**
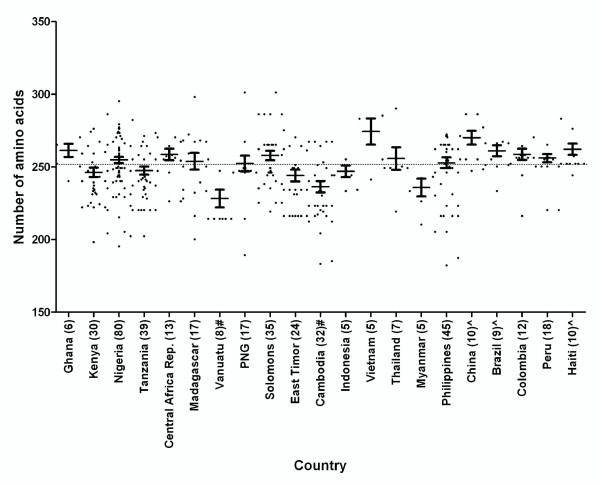
**The length of PfHRP2 (number of aa) in countries with ≥5 samples**. Numbers in brackets indicate the number of samples. The dotted line indicates the global mean. ^ The mean number is significantly higher than the global mean (p < 0.05); # the mean number is significantly lower than the global mean (p < 0.05)

The length of the deduced PfHRP2 sequence encoded by the exon 2 varies from 187 to 306 aa (average 252.2 aa, Figure 2). When compared to the global mean, the mean of amino acid length was found to be significantly lower in Vanuatu (mean = 228.3, P < 0.01) and Cambodia (mean = 236.3, P < 0.01) and significantly higher in China (mean = 270.1, P < 0.01), Brazil (mean = 261.1, P < 0.05) and Haiti (mean = 262.2, P < 0.05, Figure 2). In Vietnam (mean = 274.4) the total aa length was greater than the global mean of 252.2, however due to small sample size it was not possible to gauge statistical significance. The lowest overall variation in the number of total amino acids for *pfhrp2 *for this sample set was seen in Indonesia (range 238-260 aa), the highest variation in Papua New Guinea (PNG) (194-306 aa), Nigeria (200-300 aa), and Madagascar (203-303 aa) (Additional file 1: Table S1).

Almost all (455/458, 99.3%) deduced amino acid sequences of PfHRP2 began with the type 1 repeat and universally (100%) concluded with the type 12 repeat. While most sequences had conserved start and end amino acid repeats, the sequence varied considerably in the organisation of the repeats and the number of each repeat in the central region of the sequence. A conserved motif of repeat types 2, 3, 5, 7, 8, 2, 7 was found in the central region of the sequence in 201 of the 458 isolates sequenced (44%). In a further 38% (174/458) of the sequences part of this motif was present. A total of 83 sequences (18%) did not contain the motif.

A total of 20 different types of amino acid repeats were identified in 458 PfHRP2 sequences (Table 2). Repeat type 2 and type 12 were observed in 100% of the isolates sequenced (Additional file 2: Table S2). Types 1, 6 and 7 were found in over 97% of isolates (Additional file 2: Table S2). The prevalence of repeat types 3, 5, 8 and 10 was found to differ between the geographic areas considered: 100% in parasites from some areas and between 70% and 100% in other areas. The type 4 repeat was present in less than 50% of isolates in all regions. The remaining 10 types were limited to a few isolates, and some were limited to a cluster of countries (Additional file 2: Table S2).

**Figure 3:**
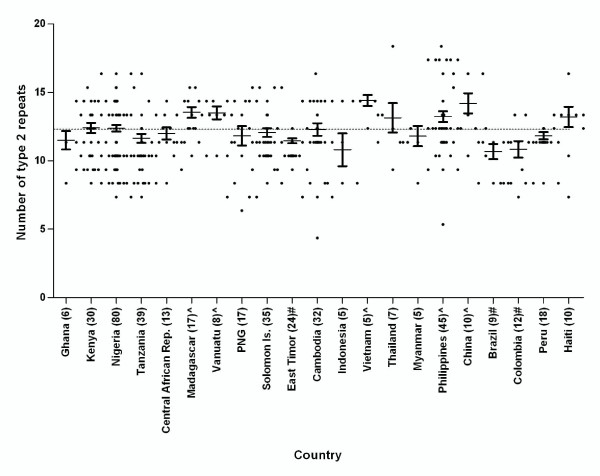
**The number of type 2 repeat present in PfHRP2 in countries with ≥ 5 samples**. Numbers in brackets indicate the number of samples. The dotted line indicates the global mean. ^ The mean number is significantly higher than the global mean (p < 0.05); # the mean number is significantly lower than the global mean (p < 0.05)

**Figure 4:**
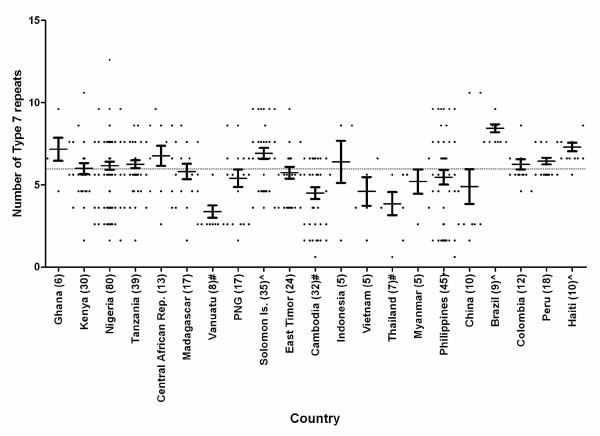
**The number of Type 7 repeat present in PfHRP2 in countries with ≥5 samples**. Numbers in brackets indicate the number of samples. The dotted line indicates the global mean. ^ The mean number is significantly higher than the global mean (p < 0.05); # the mean number is significantly lower than the global mean (p < 0.05)

With the exception of type 12 the number of each repeat type was observed to vary between different parasite isolates from the same country and between countries. When grouped by region, significant differences were found between regions for the number of repeat types 1, 2, 6 and 7 (P < 0.0001), as well as 3, 4, 5, 10, 11, 13 and 14 (P < 0.05). Further analysis was performed to examine differences in the number of types 2 and 7 repeats between countries (countries with less than 5 samples were excluded). Figures 3 and 4 illustrate the variations across different countries in the number of repeat types 2 and 7, respectively. Countries with significantly higher or lower numbers of type 2 and 7 repeats than their global means are identified and indicated in Additional file 1: Table S1.

### Variation in *pfhrp3*

The size of the *pfhrp3 *gene fragment amplified by PCR ranged from 294 bp to 552 bp, largely due to variation in numbers of 18- and 15- bp repeats. 42 different sequence types were identified from 80 isolates originating from 20 countries. Twenty-six isolates had a unique *pfhrp3 *sequence, while the remaining 54 isolates combined to give a total 16 sequence types. Of these 16 sequence types, 4 types were shared by isolates from the same country and the remaining 12 sequence types were common to isolates from different countries (Table 3). The ratio of unique over total number of sequences calculated for *pfhrp3 *was 0.525 (42/80) indicating that there is a different sequence every 1.90 parasite isolates examined for *pfhrp3*. Overall the level of diversity in *pfhrp3*, as measured by the proportion of unique sequences was significantly lower than that observed for *pfhrp2 *(P < 0.01). The lowest ratio was observed in Peru (*n *= 6, ratio 0.33), followed by Philippines (*n *= 7, ratio 0.57) and Solomon Islands (*n *= 13, ratio 0.69), a similar rank observed for *pfhrp2 *for these countries. A higher ratio was observed for PNG (*n *= 7, ratio 0.85) and Kenya (*n *= 6, ratio 1.0).

A total of nine different amino acid repeats were identified from the PfHRP3 sequences (Table 2). All deduced amino acid sequences of the PfHRP3 began with the type 1 repeat, and ended with a type 4 repeat. All sequences contained each of the nine different amino acid repeat types with varying frequency (Additional file 3: Table S3). Two sequences also contained one copy of the type 2 repeat. Most PfHRP3 sequences had one non-repetitive region while some isolates from three countries showed two non-repetitive regions (Additional file 3: Table S3). The non-repetitive region located in the centre of the sequence was identical for all sequence types, except for those sequences with two non-repetitive regions which consistently ended in a type 20 repeat. Upstream of the non-repetitive region, the sequences showed variability in the number of type 16 repeats (AHHAAN). Downstream of the non-repetitive region varied in the number of type 17 and 18 repeats in isolates from different countries (Additional file 3: Table S3).

The deduced amino acid sequence of PfHRP3 observed ranging in length from 98 to 178 aa. The lowest overall variation in the total number of amino acids for *pfhrp3 *(Additional file: 3 Table S3) for this sample set was seen in China (124-150 amino acids), the highest variation in Nigeria (114-170 amino acids), the Solomon Islands (98-184 amino acids) and PNG (104-170 amino acids). When compared with a mean of 144.5 amino acids for total global isolates, the mean amino acid length was found to be significantly higher among the samples from Peru (mean = 168.7, P < 0.01).

### PfHRP2 and PfHRP3 amino acid length correlation

To examine whether a correlation existed between amino acid length of PfHRP2 and PfHRP3, we examined the relationship of total amino acid length for both genes. No significant correlation existed between the length of *pfhrp2 *and *pfhrp3 *(P > 0.05).

### *pfhrp2 *and *pfhrp3 *deletions

Absence of the *pfhrp2 *gene was only observed in two laboratory adapted lines: Dd2 and D10 [20,27], but not in any field isolates. The lack of *pfhrp3 *gene was also only observed in a laboratory-adapted line of HB3 [17].

### Relationship between *PfHRP2 *sequence diversity and the RDT detection rate

The proportion of the 34 PfHRP2-detecting RDTs which "detected" each of the 79 parasite isolates at a density of 200 parasites/μl was calculated. In the WHO product testing, "detected" was defined as returning 4/4 positive tests against an isolate [26]. Linear regression analysis was used to investigate if there was a relationship between sequence structure of the PfHRP2 and percent detected. No significant relationship could be established between PfHRP2 structure types and percent detected (P > 0.05).

## Discussion

PfHRP2 is the target antigen for many RDTs detecting *P. falciparum*. In this study, *pfhrp2 *from 458 *P. falciparum *isolates collected from 38 countries, all major malaria endemic areas, were sequenced and evaluated. It was shown that this gene exhibits extensive diversity both within and between countries and regions. A similar protein, PfHRP3, also exhibited extensive diversity between parasite isolates examined. This comprehensive examination and analysis of the extent of the diversity and geographic variation in these genes provides important information for laboratory and field evaluation of malaria RDTs. It also provides an indication of whether the genetic diversity in the antigen contributes to the variability in RDT sensitivity.

Both *pfhrp2 *and *pfhrp3 *are located in the subtelomeric regions of chromosomes. In general, genes located in telomeric and subtelomeric regions of *Plasmodium *have vast genetic diversity, and are highly susceptible to changes during recombination events [28-33]. Subtelomeric regions from different malaria species appear to have undergone rapid evolution, with significant sequence variation generated in the complex repeats in these regions [32,33]. Molecular mechanisms contributing to the generation of tandemly repeated regions, changes in the length of repeat blocks and other variation events include slipped strand mispairing followed by DNA replication or repair, unequal reciprocal combination and gene conversion [34-39]. The different organization and varying number of repeats observed in both *pfhrp2 *and *pfhrp3 *are likely the result of frequent recombination of the chromosomes. Therefore, a correlation between malaria transmission intensity and *pfhrp2 *diversity should be expected, as malaria infections in high transmission settings often involve co-infection of multiple strains which increase the probability of recombination during sexual reproduction in the mosquito vector. Indeed, this general trend was observed in this study with the ratio of different *pfhrp2 *sequence types to total sequences being higher in countries with high transmission intensity such as in Africa, and lower in South American and Asian countries. However, this may also reflect differences in the geographical spread of collections within countries, which varied between sites.

The function of PfHRP2 still remains to be determined. Early theories suggested that PfHRP2 may be involved in detoxification of free haem by converting it to inactive haemozoin [40,41]. Other theories suggest that PfHRP2 may be involved in remodeling the infected erythrocyte cytoskeleton [42] and in modulating host immune responses [43]. The extensive diversity in the *pfhrp2 *sequence provides evidence that the function(s) of the molecule is not affected by the sequence diversity in exon 2 and that parasites with a particular *pfhrp2 *sequence do not appear to have a significant fitness or survival advantage. The host immune responses may also contribute to the maintenance of diversity by selecting for immunologicly different types.

A mechanism of chromosome breakage and healing is believed to contribute to generation of deletions in various parasite chromosomes [27]. Chromosome deletions at the *pfhrp2 *locus of chromosome 7 and *pfhrp3 *locus of chromosome 13 have been observed in laboratory-adapted lines [17,20,27], and in field isolates from the Amazon region of Peru [44]. This observation of gene deletions in field isolates suggests that PfHRP2-detecting RDTs may not be reliable for detecting *P. falciparum *infections in this region of South America, and also raised a serious question as to whether parasites lacking PfHRP2 and PfHRP3 may exist in other parts of the world and what are possible fitness or survival advantages. In the 485 isolates, collected in over 30 malaria endemic countries, that we examined, we did not observe any deletions of *pfhrp2 *or *pfhrp3 *in any field isolates, suggesting that the parasites with gene deletions may not be widespread outside of South America. However, it should be noted that the sampling did not include some endemic regions (e.g. South and Western Asia) and the number of samples examined for some countries was quite small, not coming from different areas within the country. To ensure the performance of RDTs, efforts should be taken to monitor the existence and spread of parasites with gene deletions, especially when false negative results associated with a high parasitaemia are reported.

*Pfhrp2 *and *pfhrp3 *variability appears to occur independent of the other, as no correlation between the lengths of the two genes was evident. However, both proteins share several repeats, such as type 1 and 2 repeats. These shared repeats are probably the basis for observed cross reactivity between PfHRP2 and PfHRP3 by monoclonal antibodies against PfHRP2 [8,18], which may contribute to the detection sensitivity of PfHRP2-detecting RDTs and reduce the effect of PfHRP2 variability on RDT sensitivity, particularly at high parasite densities.

The organization and the number of repeats in PfHRP2 vary extensively between parasite isolates. Theoretically, the presence and absence, as well as the number of repeats could affect the binding affinity of the antibodies used in RDTs to the parasites' antigen. Indeed, this preliminary analysis based on 16 cultured lines and tested on two earlier malaria RDTs, Paracheck Pf (Orchid Biomedical Systems, India) and ICT Malaria (ICT Diagnostics, South Africa), yielded a binary logistic regression model that was able to predict detection sensitivity of these two RDTs based on sequence structure [24]. However, the number of isolates used was limited and cultured parasites were used. In this study, the analysis was repeated using the recently completed WHO product testing of malaria RDTs (Round 1) results that included the testing of 34 PfHRP2-detecting RDTs tested against 79 isolates at 200 parasites/μl. This much larger sample size enabled more stringent analysis. The regression analysis this time did not show a correlation between PfHRP2 structure and the overall RDT detection rates. This result suggests that the performance of this group of RDTs is not greatly affected by the diversity of PfHRP2 at parasite densities of 200 parasite/μL or above and, therefore, should increase the confidence in the performance of the devices in various geographic locations. However, this does not exclude an effect of sequence diversity on RDT detection rates at lower parasite densities, or by RDTs employing other monoclonal antibodies.

## Conclusions

PfHRP2, the most common target of malaria RDTs, is highly polymorphic throughout the malaria-endemic regions included in this study. While this may affect RDT sensitivity at very low parasite densities ( < 200 parasites/μL), it appears unlikely to have a major impact in most endemic regions at parasite densities usually seen in clinical malaria (200 parasites/μL and above). The extensive diversity in the *pfhrp2 *sequence also provides evidence that there has not been a strong evolutionary selection for any particular type of sequence. The better understanding of the structure of PfHRP2 and its variation contributes to the evaluation and testing of malaria RDTs, the RDT quality assurance programmes and helps improve malaria RDTs.

## Competing interests

The authors declare that they have no competing interests.

## Authors' contributions

JB1 carried out molecular genetic studies, sequence alignment, statistical analyses, and drafted the manuscript. MFH carried out molecular genetic studies and submission of sequences to GenBank. AP and MG carried out statistical analyses. NC assisted with molecular genetic studies for the Nigerian isolates. SA, FA, JB2, AA, DB, JC, DD, DFE, DG, JH, MPK, JL, CM1, DM, CM2, SN, BO, PO, WO, SQW supervised, carried out and coordinated field collection of isolates from patients. DB, JM and QC conceived of the study, participated in its design and coordination and helped to draft the manuscript. All authors read and approved the final manuscript.

## Supplementary Material

Additional file 1 Table S1: Comparison of the length (aa) and number of each repeat in PFHRP2 in parasites from different geographical areas. Click here for file

Additional file 2 Table S2: Frequency (in percentage) of different amino acid repeats (types 1-24) observed in PfHRP2 of isolates obtained from various geographic areas and countries (sample number > 10). Click here for file

Additional file 3 Table S3: Variation of PfHRP3 sequences. Click here for file
