# Supplementary material for: Global sequence variation in the histidine-rich proteins 2 and 3 of Plasmodium falciparum: implications for the performance of malaria rapid diagnostic tests
Source: Malar J. 2010 May 17;9:129. doi: 10.1186/1475-2875-9-129 (PMC2893195; doi:10.1186/1475-2875-9-129)
Supplement: Additional file 1 — Table S1: Comparison of the length (aa) and number of each repeat in PFHRP2 in parasites from different geographical areas. [file 1475-2875-9-129-S1.DOC]

Table S1. Comparison of the length (aa) and number of each repeat in PFHRP2 in parasites from different geographical areas.

| Region | Country | n | Length (aa) | Number of individual repeat | | | | | | | | | | | | | | | | | | | |
| --- | --- | --- | --- | --- | --- | --- | --- | --- | --- | --- | --- | --- | --- | --- | --- | --- | --- | --- | --- | --- | --- | --- | --- |
|  |  |  |  | 1* | 2* | 3* | 4* | 5* | 6* | 7* | 8 | 9 | 10* | 11* | 12 | 13* | 14* | 19 | 20 | 21 | 22 | 23 | 24 |
| **Africa** | C.Africa R | 13 | 231-275 | 0-5 | 9-15 | 0-3 | 0-3 | 0-1 | 2-6 | 3-10 | 0-3 | 0 | 0-3 | 0 | 1 | 0-1 | 0-1 | 0 | 0 | 0 | 0-1 | 0 | 0-1 |
|  | Kenya | 30 | 203-281 | 1-5 | 9-16 | 0-2 | 0-2 | 0-1 | 0-7 | 2-11 | 0-2 | 0 | 0-3 | 0 | 1 | 0-1 | 0-1 | 0 | 0 | 0 | 0 | 0 | 0 |
|  | Madagascar | 17 | 205-303^ | 1-4 | 11-16 | 0-2 | 0-4 | 0-1 | 2-7 | 2-9 | 1-2 | 0 | 0-2 | 0 | 1 | 0 | 0-1 | 0 | 0 | 0 | 0 | 0 | 0 |
|  | Nigeria | 80 | 200-300^ | 0-7 | 8-17 | 0-2 | 0-3 | 0-3 | 1-7 | 2-13 | 0-3 | 0 | 0-4 | 0 | 1 | 0-2 | 0-1 | 0 | 0 | 0 | 0 | 0-1 | 0 |
|  | Tanzania | 39 | 207-287 | 0-7 | 8-17 | 0-2 | 0-2 | 0-2 | 2-6 | 2-9 | 0-3 | 0 | 0-3 | 0 | 1 | 0-1 | 0-1 | 0 | 0 | 0 | 0 | 0-1 | 0 |
|  | Others | 27 | 224-288 | 1-6 | 7-17 | 0-3 | 0-3 | 0-3 | 2-7 | 3-13 | 0-2 | 0 | 0-2 | 0 | 1 | 0-1 | 0-1 | 0 | 0 | 0 | 0 | 0 | 0 |
|  | **Sub Total** | **206** | **200-303** | **0-7** | **7-17** | **0-3** | **0-4** | **0-3** | **0-7** | **2-13** | **0-3** | **0** | **0-4** | **0** | **1** | **0-2** | **0-1** | **0** | **0** | **0** | **0-1** | **0-1** | **0-1** |
| **Southwest**  **Pacific** | East Timor | 24 | 221-280 | 2-5 | 10-13 | 1-2 | 0-1 | 1-2 | 1-6 | 4-10 | 1-2 | 0 | 0-3 | 0 | 1 | 0-1 | 0-1 | 0 | 0 | 0 | 0 | 0 | 0 |
|  | PNG | 17 | 194-306^ | 1-4 | 7-15 | 1-2 | 0-2 | 1-3 | 2-7 | 2-9 | 1-2 | 0 | 1-2 | 0 | 1 | 0 | 0-1 | 0 | 0 | 0 | 0 | 0 | 0 |
|  | Solomon Is. | 35 | 224-306 | 2-6 | 9-16 | 1-2 | 0-1 | 0-2 | 2-7 | 4-10 | 1 | 0 | 1-2 | 0-1 | 1 | 0-1 | 0-1 | 0 | 0 | 0 | 0 | 0 | 0 |
|  | Vanuatu | 8 | 219-260 | 1-4 | 11-15 | 1-2 | 0-1 | 1 | 2-6 | 3-6 | 1-2 | 0 | 1-2 | 0 | 1 | 0 | 0 | 0 | 0 | 0 | 0 | 0 | 0 |
|  | **Sub Total** | **84** | **194-306** | **1-6** | **7-16** | **1-2** | **0-2** | **0-3** | **1-7** | **2-10** | **1-2** | **0** | **0-3** | **0-1** | **1** | **0-1** | **0-1** | **0** | **0** | **0** | **0** | **0** | **0** |
| **Central**  **&**  **South America** | Brazil | 9 | 238-272 | 1-4 | 9-13 | 1-2 | 0-1 | 0-2 | 2-5 | 8-10 | 0-2 | 0 | 0-3 | 0 | 1 | 0 | 0 | 0 | 0 | 0 | 0 | 0 | 0 |
|  | Colombia | 12 | 221-275# | 1-7 | 8-14 | 0-1 | 0-2 | 0-2 | 2-7 | 5-9 | 1-2 | 0 | 1-2 | 0 | 1 | 0-1 | 0 | 0 | 0-1 | 0-1 | 0 | 0 | 0 |
|  | Haiti | 10 | 249-288 | 2-4 | 8-17 | 0-2 | 0-2 | 0-2 | 2-7 | 6-9 | 1-2 | 0 | 0-2 | 0 | 1 | 0-2 | 0 | 0 | 0 | 0 | 0 | 0 | 0 |
|  | Peru | 18 | 225-270 | 1-5 | 9-13 | 0-2 | 0-1 | 0-2 | 2-7 | 6-8 | 1-2 | 0 | 1-2 | 0 | 1 | 0-1 | 0 | 0 | 0 | 0 | 0 | 0-1 | 0 |
|  | Others | 8 | 249-272 | 1-5 | 12-13 | 1-2 | 0-1 | 1 | 2-5 | 7-8 | 1-2 | 0 | 1-2 | 0 | 1 | 0 | 0 | 0 | 0 | 0 | 0 | 0 | 0 |
|  | **Sub Total** | **57** | **221-288** | **1-7** | **8-17** | **0-2** | **0-2** | **0-2** | **2-7** | **5-10** | **0-2** | **0** | **0-3** | **0** | **1** | **0-2** | **0** | **0** | **0-1** | **0-1** | **0** | **0-1** | **0** |
| **Asia** | Cambodia | 32 | 188-272 | 1-4 | 5-17 | 0-3 | 0-2 | 0-2 | 2-5 | 1-7 | 0-2 | 0 | 0-3 | 0 | 1 | 0 | 0-1 | 0 | 0-1 | 0-1 | 0-1 | 0-1 | 0 |
|  | China | 10 | 252-291 | 2-5 | 11-17 | 1-2 | 0-3 | 0-1 | 2-6 | 2-11 | 1-2 | 0 | 1-3 | 0 | 1 | 0-1 | 0 | 0 | 0 | 0 | 0 | 0 | 0 |
|  | Indonesia | 5 | 238-260 | 1-3 | 9-15 | 1-3 | 0-2 | 1-2 | 3-7 | 2-9 | 1-2 | 0 | 0-2 | 0 | 1 | 0 | 0 | 0 | 0 | 0 | 0 | 0 | 0 |
|  | Myanmar | 5 | 215-248 | 1-3 | 9-13 | 1-2 | 0-2 | 1-2 | 2-3 | 3-7 | 1-2 | 0 | 0-2 | 0 | 1 | 0 | 0-1 | 0 | 0 | 0 | 0 | 0 | 0 |
|  | Philippines | 45 | 187-277 | 1-4 | 6-19 | 0-3 | 0-3 | 0-2 | 2-7 | 0-10 | 0-3 | 0-1 | 0-2 | 0-1 | 1 | 0 | 0-1 | 0-1 | 0 | 0 | 0 | 0 | 0 |
|  | Vietnam | 5 | 246-290 | 2-6 | 13-15 | 1-2 | 1-2 | 1 | 3-4 | 2-7 | 1 | 0 | 1-2 | 0 | 1 | 0 | 0 | 0 | 0 | 0 | 0 | 0 | 0 |
|  | Thailand | 7 | 224-295 | 1-5 | 10-19 | 1-3 | 0-2 | 0-1 | 2-5 | 1-6 | 0-2 | 0 | 0-2 | 0 | 1 | 0-1 | 0-1 | 0 | 0 | 0 | 0 | 0 | 0 |
|  | Others | 2 | 216-235 | 3 | 9-13 | 1-2 | 0 | 0-1 | 2-4 | 6-7 | 1 | 0 | 0-1 | 0 | 1 | 0 | 0 | 0 | 0 | 0 | 0 | 0 | 0 |
|  | **Sub Total** | **111** | **187-291** | **1-6** | **5-19** | **0-3** | **0-3** | **0-2** | **2-7** | **0-11** | **0-3** | **0-1** | **0-3** | **0-1** | **1** | **0-1** | **0-1** | **0-1** | **0-1** | **0-1** | **0-1** | **0-1** | **0** |
| **Global Total** | | **458** | **187-306** | **0-7** | **5-19** | **0-3** | **0-4** | **0-3** | **0-7** | **0-13** | **0-3** | **0-1** | **0-4** | **0-1** | **1** | **0-2** | **0-1** | **0-1** | **0-1** | **0-1** | **0-1** | **0-1** | **0-1** |

Note: * The mean number of this repeat is significantly different across different areas (p<0.05).

^ The range is significantly higher than the global range (p<0.05).

# The range is significantly lower than the global range (p<0.05).
