# Supplementary material for: Global sequence variation in the histidine-rich proteins 2 and 3 of Plasmodium falciparum: implications for the performance of malaria rapid diagnostic tests
Source: Malar J. 2010 May 17;9:129. doi: 10.1186/1475-2875-9-129 (PMC2893195; doi:10.1186/1475-2875-9-129)
Supplement: Additional file 2 — Table S2: Frequency (in percentage) of different amino acid repeats (types 1-24) observed in PfHRP2 of isolates obtained from various geographic areas and countries (sample number > 10). [file 1475-2875-9-129-S2.DOC]

Table S2. Frequency (in percentage) of different amino acid repeats (types 1-24) observed in PfHRP2 of isolates obtained from various geographic areas and countries (sample number >10).

| Area | n | Repeats | | | | | | | | | | | | | | | | | | | |
| --- | --- | --- | --- | --- | --- | --- | --- | --- | --- | --- | --- | --- | --- | --- | --- | --- | --- | --- | --- | --- | --- |
|  |  | 1 | 2 | 3 | 4 | 5 | 6 | 7 | 8 | 9 | 10 | 11 | 12 | 13 | 14 | 19 | 20 | 21 | 22 | 23 | 24 |
| Africa | 206 | 98.5 | 100 | 88.3 | 25.7 | 74.2 | 99.5 | 100 | 92.7 | 0 | 89.8 | 0 | 100 | 9.7 | 11.2 | 0 | 0 | 0 | 0.5 | 3.4 | 0.4 |
| SE Asia | 101 | 100 | 100 | 90.1 | 38.7 | 84.2 | 100 | 99.1 | 92.3 | 0.9 | 83.8 | 0.9 | 100 | 1.8 | 3.6 | 1.8 | 1.8 | 1.8 | 0.9 | 0.9 | 0 |
| Cambodia | 32 | 100 | 100 | 78.0 | 38.0 | 87.0 | 100 | 100 | 90.6 | 0 | 75.0 | 0 | 100 | 0 | 3.0 | 0 | 6.0 | 6.0 | 3.0 | 3.0 | 0 |
| China | 10 | 100 | 100 | 100 | 50.0 | 90.0 | 100 | 100 | 100 | 0 | 100 | 0 | 100 | 10 | 0 | 0 | 0 | 0 | 0 | 0 | 0 |
| Philippines | 45 | 100 | 100 | 91.1 | 26.6 | 75.5 | 100 | 97.7 | 91.1 | 2.2 | 88.8 | 2.2 | 100 | 0 | 2.2 | 4.4 | 0 | 0 | 0 | 0 | 0 |
| Southwest Pacific | 84 | 100 | 100 | 100 | 21.5 | 91.6 | 100 | 100 | 100 | 0 | 98.8 | 4.7 | 100 | 3.5 | 13.1 | 0 | 0 | 0 | 0 | 0 | 0 |
| East Timor | 24 | 100 | 100 | 100 | 12.5 | 100 | 100 | 100 | 100 | 0 | 95.8 | 0 | 100 | 4.1 | 29.2 | 0 | 0 | 0 | 0 | 0 | 0 |
| PNG | 17 | 100 | 100 | 100 | 35.3 | 100 | 100 | 100 | 100 | 0 | 100 | 0 | 100 | 0 | 17.6 | 0 | 0 | 0 | 0 | 0 | 0 |
| Solomon Is. | 35 | 100 | 100 | 100 | 22.8 | 80.0 | 100 | 100 | 100 | 0 | 100 | 11.4 | 100 | 5.7 | 2.8 | 0 | 0 | 0 | 0 | 0 | 0 |
| Central and South America | 57 | 100 | 100 | 82.5 | 28.1 | 78.9 | 100 | 100 | 98.2 | 0 | 92.9 | 0 | 100 | 8.7 | 0 | 0 | 1.7 | 1.7 | 0 | 3.5 | 0 |
| OVERALL | 458 | 99.3 | 100 | 90.2 | 28.3 | 80.6 | 99.7 | 99.7 | 94.7 | 0.2 | 90.2 | 1.1 | 100 | 6.7 | 8.5 | 0.4 | 0.7 | 2.2 | 0.2 | 2.2 | 0.4 |
