# Supplementary material for: Global sequence variation in the histidine-rich proteins 2 and 3 of Plasmodium falciparum: implications for the performance of malaria rapid diagnostic tests
Source: Malar J. 2010 May 17;9:129. doi: 10.1186/1475-2875-9-129 (PMC2893195; doi:10.1186/1475-2875-9-129)
Supplement: Additional file 3 — Table S3: Variation of PfHRP3 sequences. [file 1475-2875-9-129-S3.DOC]

Table S3. Variation of PfHRP3 sequences.

| Region | Country | *n* | unique/total seq ratio (n> 4) | Length (aa)  Mean (range) | Repeats (mean number) | | | | | | | | | Non-repetitive region | |
| --- | --- | --- | --- | --- | --- | --- | --- | --- | --- | --- | --- | --- | --- | --- | --- |
|  |  |  |  |  | 1 | 2 | 4 | 7 | 15 | 16 | 17 | 18 | 20 | 1 | 2 |
| AFRICA | Central Africa Rep. | 1 |  | 138 | 1 | 0 | 1 | 1 | 1 | 11 | 6 | 2 | 1 | 1 |  |
|  | Gambia | 1 |  | 165 | 1 | 0 | 1 | 1 | 1 | 13 | 7 | 4 | 1 | 1 |  |
|  | Nigeria | 16 | 0.68 | 149 (114-170) | 1.1# | 0 | 1.1 | 1 | 0.9# | 12.1^ | 6.5 | 2.4 | 1 | 1 |  |
|  | Kenya | 6 | 1.0 | 152 (135-171) | 1.5 | 0 | 1 | 1 | 1 | 12.1 | 6.5 | 2.2 | 1 | 1 |  |
|  | Sierra Leone | 1 |  | 152 | 1 | 0 | 1 | 1 | 0 | 14 | 7 | 2 | 1 | 1 |  |
|  | Tanzania | 2 |  | 153 (150-156) | 1 | 0 | 1 | 1 | 1 | 11.5 | 7 | 2 | 1 | 1 |  |
|  | Sub total | 27 |  |  |  |  |  |  |  |  |  |  |  |  |  |
| Central & South America | Brazil | 2 |  | 150 | 1 | 0 | 1 | 1 | 1 | 13 | 6 | 2 | 1 | 1 |  |
|  | Colombia | 1 |  | 171 | 1 | 0 | 1 | 1 | 1 | 14 | 7 | 4 | 1 | 1 |  |
|  | Haiti | 4 |  | 149 (126-173) | 1 | 0 | 1 | 1 | 1 | 12.2 | 6.3 | 2.5 | 1 | 1 |  |
|  | Peru | 6 | 0.33 | 168 (150-178) ^ | 1 | 0 | 1 | 1 | 1 | 15^ | 7.3 | 2 | 1 | 1 |  |
|  | Suriname | 2 |  | 148 | 2 | 0 | 1 | 1 | 1 | 12 | 5 | 2 | 1 | 1 |  |
|  | Sub total | 15 |  |  |  |  |  |  |  |  |  |  |  |  |  |
| SOUTH-West Pacific | Papua New Guinea | 7 | 0.85 | 127 (104-170) | 1.4 | 0 | 1 | 1.1 | 1.1 | 8.1 | 6.2 | 2.3 | 1.1 | 1 |  |
|  | Solomon Is. | 13 | 0.69 | 132 (98-184) | 1.5 | 0 | 1 | 1.2 | 1.3 | 7.1# | 6.9 | 2.4 | 1.3 | 1 | 2 |
|  | Vanuatu | 1 |  | 114 | 1 | 0 | 1 | 1 | 1 | 7 | 7 | 1 | 1 | 1 |  |
|  | Sub total | 21 |  |  |  |  |  |  |  |  |  |  |  |  |  |
| Asia | Malaysia | 1 |  | 138 | 1 | 0 | 1 | 1 | 1 | 11 | 6 | 2 | 1 | 1 |  |
|  | Thailand | 4 |  | 151 (134-171) | 1 | 0 | 1 | 1 | 1 | 12.8 | 6 | 2.5 | 1 | 1 |  |
|  | Cambodia | 1 |  | 119 | 1 | 0 | 1 | 1 | 1 | 7 | 7 | 2 | 1 | 1 |  |
|  | China | 4 |  | 137 (124-150) | 1.5 | 0 | 1 | 1.2 | 1.2 | 10.2 | 5.7 | 2.2 | 1.2 | 1 | 2 |
|  | Philippines | 7 | 0.57 | 139 (112-158) | 1.5 | 0.3 | 1.2 | 1.3 | 1.3 | 8 | 5.2# | 3.6^ | 1.6 | 1 | 2 |
|  | Subtotal | 17 |  |  |  |  |  |  |  |  |  |  |  |  |  |
|  | GLOBAL TOTAL | 80 |  |  |  |  |  |  |  |  |  |  |  |  |  |

Note: ^ The mean number or the range is significantly higher than the global mean or range (p<0.05). # The mean number or the range is significantly lower than the global mean or range (p<0.05).
